# Solid-phase synthesis and pathological evaluation of pyroglutamate amyloid-β_3-42_ peptide

**DOI:** 10.1038/s41598-022-26616-x

**Published:** 2023-01-10

**Authors:** Illhwan Cho, HeeYang Lee, Donghee Lee, In Wook Park, Soljee Yoon, Hye Yun Kim, YoungSoo Kim

**Affiliations:** 1 Department of Pharmacy, College of Pharmacy, Yonsei University, Incheon, 21983 Republic of Korea; 2 College of Pharmacy, Yonsei Institute of Pharmaceutical Sciences, Yonsei University, Incheon, 21983 Republic of Korea; 3 Department of Integrative Biotechnology and Translational Medicine, Yonsei University, Incheon, 21983 Republic of Korea; 4 Yonsei-POSTECH Campus, Pohang University of Science and Technology (POSTECH), Pohang, Gyeongbuk 37673 Republic of Korea

**Keywords:** Peptides, Alzheimer's disease, Cognitive neuroscience

## Abstract

Pyroglutamate amyloid-β_3-42_ (Aβ_pE3-42_) is an N-terminally truncated and pyroglutamate-modified Aβ peptide retaining highly hydrophobic, amyloidogenic, and neurotoxic properties. In Alzheimer’s disease (AD) patients, Aβ_pE3-42_ peptides accumulate into oligomers and induce cellular toxicity and synaptic dysfunction. Aβ_pE3-42_ aggregates further seed the formation of amyloid plaques, which are the pathological hallmarks of AD. Given that Aβ_pE3-42_ peptides play critical roles in the development of neurodegeneration, a reliable and reproducible synthetic access to these peptides may support pathological and medicinal studies of AD. Here, we synthesized Aβ_pE3-42_ peptides through the microwave-assisted solid-phase peptide synthesis (SPPS). Utilizing thioflavin T fluorescence assay and dot blotting analysis with anti-amyloid oligomer antibody, the amyloidogenic activity of synthesized Aβ_pE3-42_ peptides was confirmed. We further observed the cytotoxicity of Aβ_pE3-42_ aggregates in cell viability test. To examine the cognitive deficits induced by synthetic Aβ_pE3-42_ peptides, Aβ_pE3-42_ oligomers were intracerebroventricularly injected into imprinting control region mice and Y-maze and Morris water maze tests were performed. We found that Aβ_pE3-42_ aggregates altered the expression level of postsynaptic density protein 95 in cortical lysates. Collectively, we produced Aβ_pE3-42_ peptides in the microwave-assisted SPPS and evaluated the amyloidogenic and pathological function of the synthesized peptides.

## Introduction

Alzheimer’s disease (AD) is a progressive neurodegenerative disorder accompanied by amyloid plaques in the brain^1^. The major components of plaques are amyloid-β (Aβ) peptides varying in length or N-/C-terminal modifications. One of the most abundant N-terminal truncated Aβ variants is the pyroglutamate Aβ_3-42_ (Aβ_pE3-42_) peptide, which constitutes about a quarter of total Aβ in the plaques^2,3^. Aβ_pE3-42_ is generated through a multistep protein modification starting from the sequential enzymatic cleavage of amyloid precursor protein to release Aβ_1-42_^4^. Then, the two N-terminal amino acids, aspartic acid and alanine, of Aβ_1-42_ are removed by amino peptidase A and dipeptidyl peptidase 4^5,6^. Lastly, the truncated Aβ_3-42_ peptide reacts with glutaminyl cyclase (QC), which converts the glutamate at the third position of N-terminus into a pyroglutamate^7^. The formation of a lactam ring from the pyroglutamate residue increases hydrophobicity, stability, and aggregation propensity of the Aβ_pE3-42_ peptide compared to other Aβ variants^8^. In AD brains, Aβ_pE3-42_ peptides assemble into soluble oligomers inducing cognitive dysfunction and cellular toxicity^9,10^. Aβ_pE3-42_ oligomers are resistant to the degradation by peptidases and maintain stable β-sheet formation in the aqueous media^11^. Insoluble Aβ_pE3-42_ aggregates further accelerate the formation of plaques in AD patients^12^. Recent clinical trials of an antibody drug candidate targeting Aβ_pE3-42_ aggregates have provided the evidence that Aβ_pE3-42_ is associated with amyloid plaques and cognitive impairments^13^. Thus, reproducible synthetic access to Aβ_pE3-42_ peptide is essential for biochemical, neuropathological, and medicinal studies of AD. Unlike the full-length Aβ isomers, Aβ_1-40_ and Aβ_1-42_, the production of Aβ_pE3-42_ peptides requires extra processes including removal of two N-terminal amino acids and cyclization of glutamate when acquired recombinantly^14^.

Here, we introduce a facile synthetic method of Aβ_pE3-42_ production utilizing microwave-assisted solid-phase peptide synthesis (SPPS)^15^. To test aggregation propensity of the synthesized Aβ_pE3-42_ peptides, we used in vitro assays, thioflavin T (ThT) assay and dot blotting analysis with anti-amyloid oligomer antibody. We tested the cellular toxicity of the synthesized Aβ_pE3-42_ peptides in the cell viability assay. We then intracerebroventricularly injected Aβ_pE3-42_ oligomers into imprinting control region (ICR) mouse models and performed behavior tests, Morris water maze and Y-maze tests, to identify cognitive deficits induced by synthesized Aβ_pE3-42_ peptides. We further obtained brain lysates from the Aβ_pE3-42_-infused mouse models and analyzed the alteration of the synaptic protein marker levels by the western blot method.

## Results and discussion

### Synthesis of Aβ_pE3-42_ peptides

**Figure 1:**
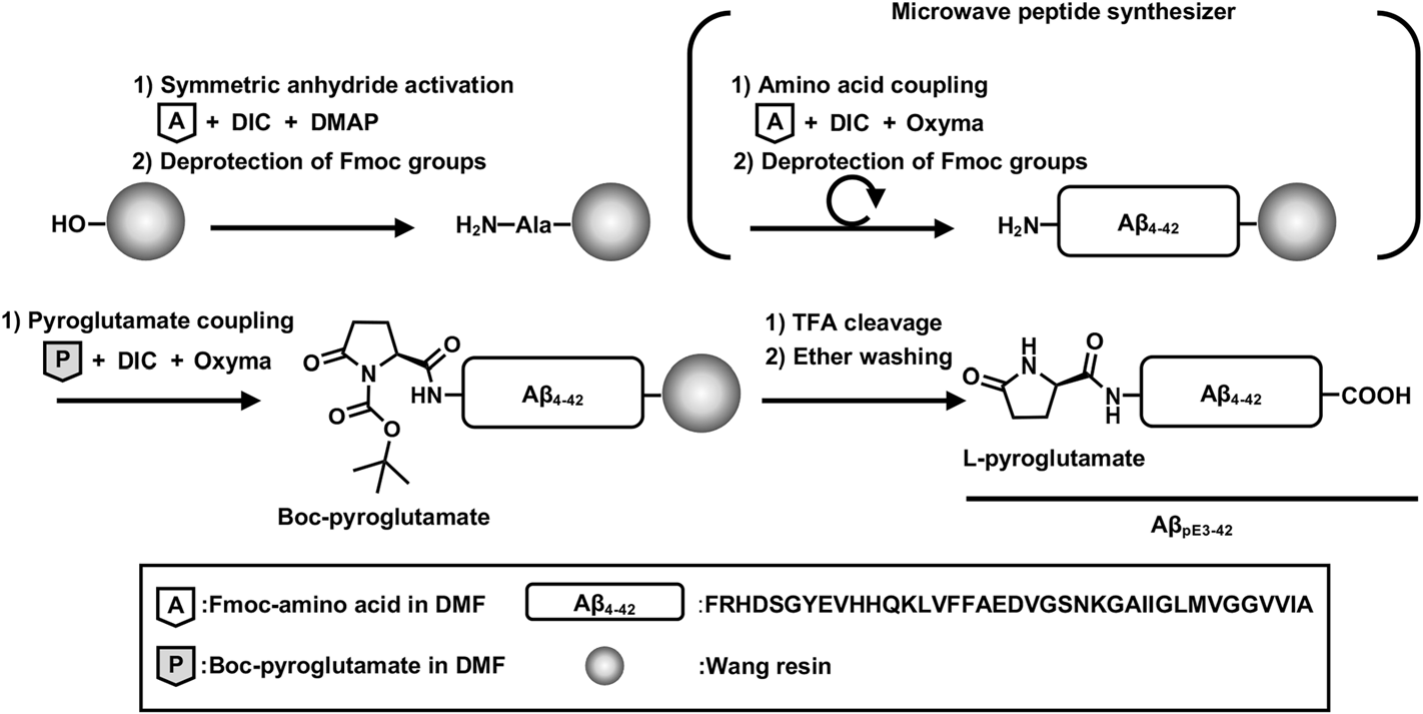
Scheme of Aβ_pE3-42_ synthesis. A graphical scheme of Aβ_pE3-42_ synthesis utilizing microwave-assisted SPPS. DMF, Dimethylformamide; Aβ_pE3-42_, Pyroglutamate amyloid-β_3-42_; Aβ_4-42_, Amyloid-β_4-42_; Boc, Tert-butoxycarbonylation; DIC, *N*,*N*′-diisopropylcarbodiimide; DMAP, 4-dimethylaminopyridine; Oxyma, Ethyl cyanohydroxylminoacetate; TFA, Trifluoroacetic acid; Fmoc, 9-fluorenylmethyloxycarbonylation; Ala, Alanine.

Although Aβ_pE3-42_ peptide is emerging as an attractive target for AD research, an effective synthetic method of this peptide is not yet developed. For the facile and reproducible production of Aβ_pE3-42_, we utilized previously-reported Aβ synthesis protocol using a microwave synthesizer for the preparation of Aβ_pE3-42_ peptide^16^. Microwave-assisted SPPS improves the efficiency of the peptide synthesis due to reduce the reaction time and to suppresses the intermolecular aggregation, resulting in the inaccessibility of the coupling reagents to react with the peptide^17,18^. Compared to the general approach of SPPS, microwave heating method increases the peptide yield from 20 to 70% and reduces the amino acid coupling time to 5 min (min)^15^. In microwave-assisted SPPS, we synthesized Aβ_pE3-42_ peptide in 6.5 h (h) and obtained 71% peptide yield. This result showed that our synthetic approach minimizes the reaction time and enhances the peptide yield of Aβ_pE3-42_. To conjugate the first C-terminal amino acids to the resin, we used the symmetric anhydride activation, and the following amino acids were sequentially synthesized to produce N-terminally truncated Aβ_4-42_ peptides and additionally coupled pyroglutamates (Fig. 1). This approach brings in benefits to avoid complicated N-terminal cleavage and glutamate cyclization steps^14^. We further purified Aβ_pE3-42_ peptides adapting reverse phase-high performance liquid chromatography analysis (Supplementary Fig. S1).

### Aggregation properties of synthetic Aβ_pE3-42_

**Figure 2:**
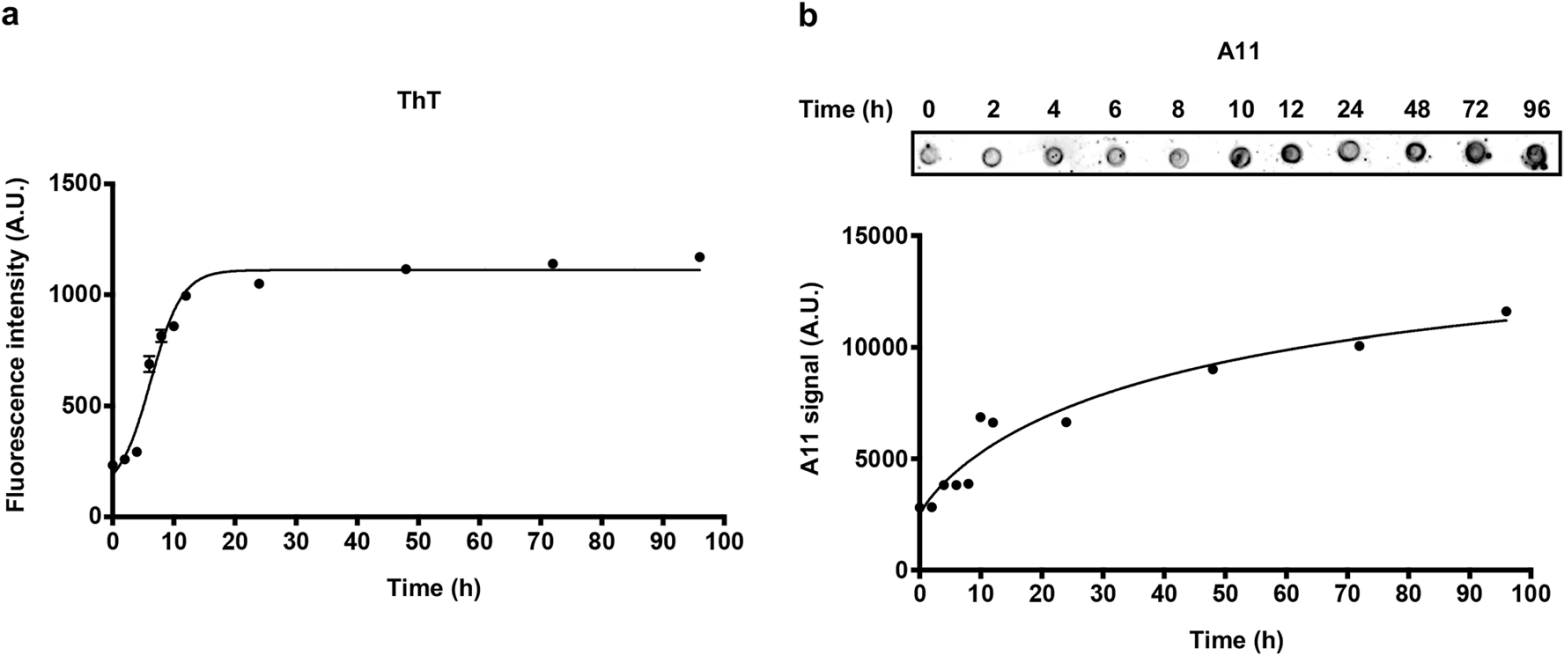
ThT fluorescence assay and dot blotting analysis of Aβ_pE3-42_ aggregation. Aβ_pE3-42_ (25 μM) samples were incubated for the time points (0, 2, 4, 6, 8, 10, 12, 24, 48, 72, 96 h) at 37 $^\circ$C. (**a**) Each of incubated Aβ_pE3-42_ sample was interacted with ThT and the fluorescence intensity was measured by the fluorescence microplate reader. (**b**) Utilizing anti-oligomer polyclonal A11, dot blotting and densitometric analysis were performed to detect oligomeric forms of incubated Aβ_pE3-42_ samples. The original dot blots and uncropped full-membrane results with A11 are presented in Supplementary Figure S4. The error bars represent S.E.M. Aβ_pE3-42_, Pyroglutamate Aβ_3-42_; h, Hours; A.U., Arbitrary units.

During the progression of AD, Aβ monomers aggregate into soluble oligomers and further become insoluble fibrils, the main components of the cerebral plaques in the brains^19^. As the misfolding and assembly is the key pathological feature of Aβ in AD, it is critical to evaluate the aggregation properties of synthetically obtained peptides. Thus, to identify the aggregation propensity of the synthesized Aβ_pE3-42_ peptide, we performed ThT fluorescence assay. ThT intercalates into the β-sheet formation of Aβ fibrils, and the shifted fluorescence intensity of ThT upon β-sheet was measured to analyze the amounts of Aβ fibrils^20^. We prepared 25 μM of Aβ_pE3-42_ samples in deionized water with 5.5% dimethyl sulfoxide (DMSO) and incubated during the time range from 0 to 96 h at 37 $^\circ$C. ThT solution (5 μM) was added to each of incubated Aβ solution samples and measured the intensity of ThT (Fig. 2a). We observed a saturation phase at 19 h of incubation, indicating the formation of β-sheet-rich Aβ_pE3-42_ fibrils. We further compared the aggregation propensity of the synthesized Aβ_pE3-42_ peptide with that of Aβ_1-42_ and Aβ_4-42_ peptides, which rapidly form aggregates^21^. Both of Aβ_1-42_ and Aβ_4-42_ peptides were produced through the previously reported synthesis protocol (Supplementary Fig. S2)^16^. In ThT assay, Aβ_pE3-42_ formed β-sheet rich fibril after 19 h of incubation time, whereas Aβ_1-42_ and Aβ_4-42_ aggregated into fibrils after 23 and 28 h of incubation, respectively (Supplementary Fig. S3). This result was consistent with the previous report that N-terminal deletion or modification of Aβ_1-42_ enhances the aggregation of N-terminal truncated Aβ peptide compared to the Aβ_1-42_^21^. As ThT assay is limited to detect β-sheet-rich fibrils, we performed dot blotting analysis using anti-amyloid oligomer A11 antibody for monitoring the formation of synthesized Aβ_pE3-42_ oligomers^22^. We prepared 2 μL of identical Aβ_pE3-42_ samples used in ThT assay and loaded on the nitrocellulose membrane and performed dot blotting analysis (Fig. 2b). The levels of Aβ_pE3-42_ oligomers were gradually increased until 96 h incubation. Interestingly, we observed oligomers even before the incubation at 37 $^\circ$C (0 h incubation sample). This result supports the previous study that monomeric Aβ_pE3-42_ sample has aggregates due to the spontaneous aggregation process of the peptide^23^. Overall, we examined the amyloidogenic properties of the synthesized Aβ_pE3-42_ peptides in both of ThT assay and dot blotting analysis.

### Cell cytotoxicity induced by synthetic Aβ_pE3-42_

**Figure 3:**
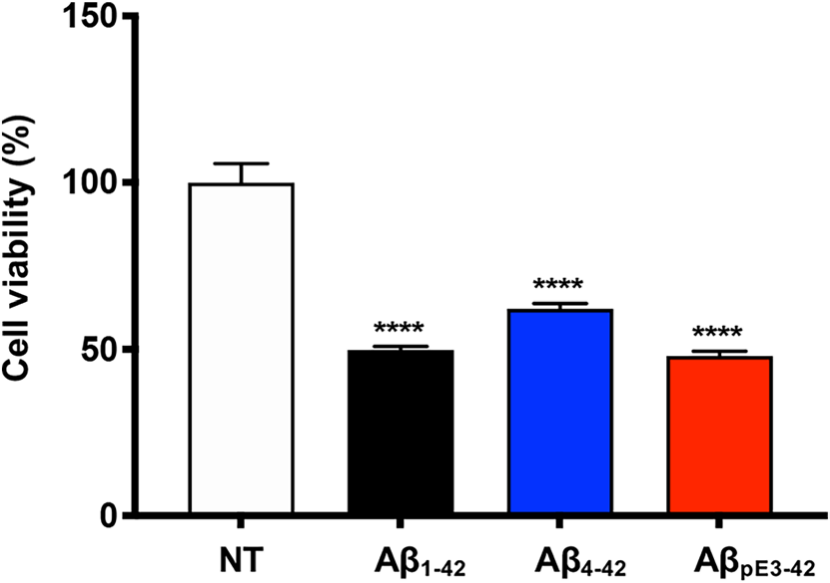
Cellular toxicity of synthesized Aβ_pE3-42_ peptide. Synthesized Aβ variants (Aβ_1-42_, Aβ_4-42_, Aβ_pE3-42_) were prepared and each of aggregated Aβ variant was treated in the cultured HT22 cell. After the treatment, the cell viability was measured utilizing MTT assay. Cell viability of Aβ variants (Aβ_1-42_, Aβ_4-42_, Aβ_pE3-42_) were statistically compared to non-treated group (4 groups, n = 3 per group). One-way ANOVA analysis followed by Bonferroni’s *post-hoc* comparison tests were performed in all statistical analyses (****p < 0.0001). The error bars represent S.E.M. NT, Non-treated; Aβ_1-42_, Amyloid-β_1-42_; Aβ_4-42_, Amyloid-β_4-42_; Aβ_pE3-42_, Pyroglutamate amyloid-β_3-42_.

To investigate the cellular toxicity of the synthesized Aβ_pE3-42_ peptide, we treated Aβ_pE3-42_ aggregates in HT22 cells (mouse hippocampal neuronal cell line). For the controls, we used Aβ_1-42_ and Aβ_4-42_ peptides, which induce cytotoxicity in neuronal cells^21^. To prepare the aggregated Aβ variants (Aβ_1-42_, Aβ_4-42_, Aβ_pE3-42_), each Aβ variant (10 μM) was incubated for 8 h at 37 $^\circ$C. After seeding the cultured HT22 cells on a 96-well plate, we treated each incubated Aβ variant for 24 h at 37 $^\circ$C. We quantified the level of cell viability using 3-(4,5-dimethylthiazol-2-yl)-2,5-diphenyltetrazolium bromide (MTT) assay (Fig. 3)^24^. Cell viability of Aβ_1-42_, Aβ_4-42_, and Aβ_pE3-42_ groups were statistically compared to non-treated group (n = 3 per group). For the statistical analysis, we performed one-way ANOVA analysis followed by Bonferroni’s *post-hoc* comparison tests. Compared to the non-treated group, the cell viability was decreased to 48.1% (*p* < 0.0001) in Aβ_pE3-42_-treated group, 49.9% (*p* < 0.0001) in Aβ_1-42_-treated group, and 62.3% (*p* < 0.0001) in Aβ_4-42_-treated group. This result showed that all the Aβ variants initiates cellular toxicity in the aggregated form. In cell viability assay, we confirmed that the synthetic Aβ_pE3-42_ aggregates induce severe cellular toxicity in mouse hippocampal neuronal cells.

### Cognitive impairments induced by synthetic Aβ_pE3-42_ in rodents

**Figure 4:**
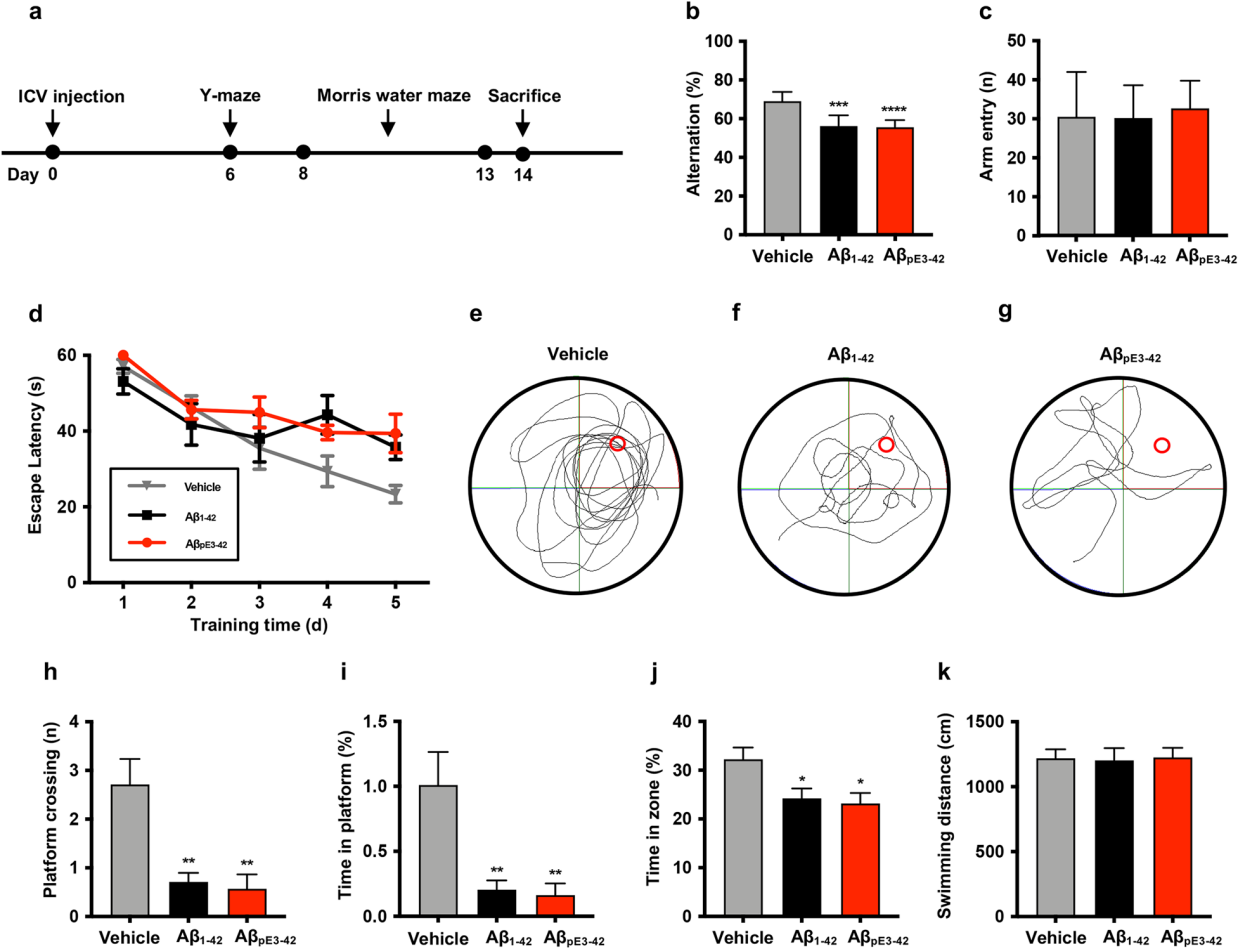
Y-maze and Morris water maze tests. (**a**) After 5 μL ICV injection of saline, Aβ_pE3-42_ (10 μM, 10% DMSO in saline) and Aβ_1-42_ (10 μM, 10% DMSO in saline) into seven-week-old male ICR mice (3 groups, n = 7 per group), Y-maze and Morris water maze tests were performed. The data from Y-maze test indicated (**b**) the alteration percentages and (**c**) total arm entries of all groups. The results from Morris water maze test showed that (**e–g**) the representative motion tail, (**h**) crossing platform, (**i**) time in platform, time in target zone (**j**), and (**k**) total swimming distance of all groups. All data were statistically compared to vehicle group. One-way ANOVA analysis followed by Bonferroni’s *post-hoc* comparison tests were performed in all statistical analyses (*p < 0.05, **p < 0.01, ***p < 0.001, ****p < 0.0001). The error bars represent S.E.M. Aβ_pE3-42_, Pyroglutamate amyloid-β_3-42_; Aβ_1-42_, Amyloid-β_1-42_; s, Seconds; d, Days.

To confirm the neuropathological function of the synthesized Aβ_pE3-42_ peptide, we injected Aβ_pE3-42_ oligomers into the lateral ventricular region of ICR mice as injected soluble Aβ oligomers inducing neurotoxicity and cognitive decline^25^. For comparison, we used Aβ_1-42_ peptide, one of the major pathological forms of Aβ variants associated with cognitive decline^26^, as a control. Aβ_1-42_ peptide was synthesized and purified through the previously reported protocol^16^. Since the dot blotting analysis result showed that soluble oligomers were clearly appeared after 8 h of incubation, we incubated 25 μM of Aβ solution for 8 h at 37 $^\circ$C to prepare oligomeric samples for the injection. We conducted 5 μL intracerebroventricular (ICV) injections of blank saline, Aβ_pE3-42_ oligomer solution (10 μM), and Aβ_1-42_ oligomer solution (10 μM) into brains of seven-week-old male ICR mice. After five days of recovery time, we performed learning and memory behavior tests, Y-maze and Morris water maze tests (Fig. 4a). All results from behavior tests were statistically compared to vehicle group (n = 7 per group). For the statistical analysis of the behavior tests, one-way ANOVA analysis followed by Bonferroni’s *post-hoc* comparison tests were performed. Y-maze is widely applied to measure the short-term memory function of mouse models due to they normally explore new arm entries of the maze rather than the arm that was previously entered^27^. We found that both of Aβ_pE3-42_ (*p* < 0.0001) and Aβ_1-42_ (*p* < 0.001) oligomers induced short-term memory impairments compared to vehicle (saline-injected) groups as the alternation levels were decreased (Fig. 4b). Total arm entry data showed that there were no significant differences of arm entries among all groups (Fig. 4c). After the Y-maze test, we performed Morris water maze test to examine the spatial learning dysfunction by Aβ_pE3-42_ oligomers^28^. During the five days of practice, we placed mice on four different quadrants in the water tank and measured time to reach the hidden platform for surviving in the water tank. In the trial day, we removed the platform and measured the crossing number, time in platform location, and time in zone. Compared to Aβ_pE3-42_ and Aβ_1-42_ groups, the escape latency of the vehicle groups was reduced on the fourth day of training (Fig. 4d). On the probe trial day, platform crossing number and time in platform of Aβ_pE3-42_ (*p* < 0.01) and Aβ_1-42_ groups (*p* < 0.01) were decreased comparing to the vehicle groups (Fig. 4e–i). We also analyzed the time in target quadrant and found that saline groups stay in the target quadrant longer than Aβ_pE3-42_ (*p* < 0.05) and Aβ_1-42_ groups (*p* < 0.05) (Fig. 4j). There were no significant differences of swimming distance among all groups (Fig. 4k). Overall, results from both of Y-maze and Morris water maze tests showed that the synthesized Aβ_pE3-42_ aggregates induced impaired short-term and spatial memory function of mice.

### Synaptic protein alteration by synthetic Aβ_pE3-42_

**Figure 5:**
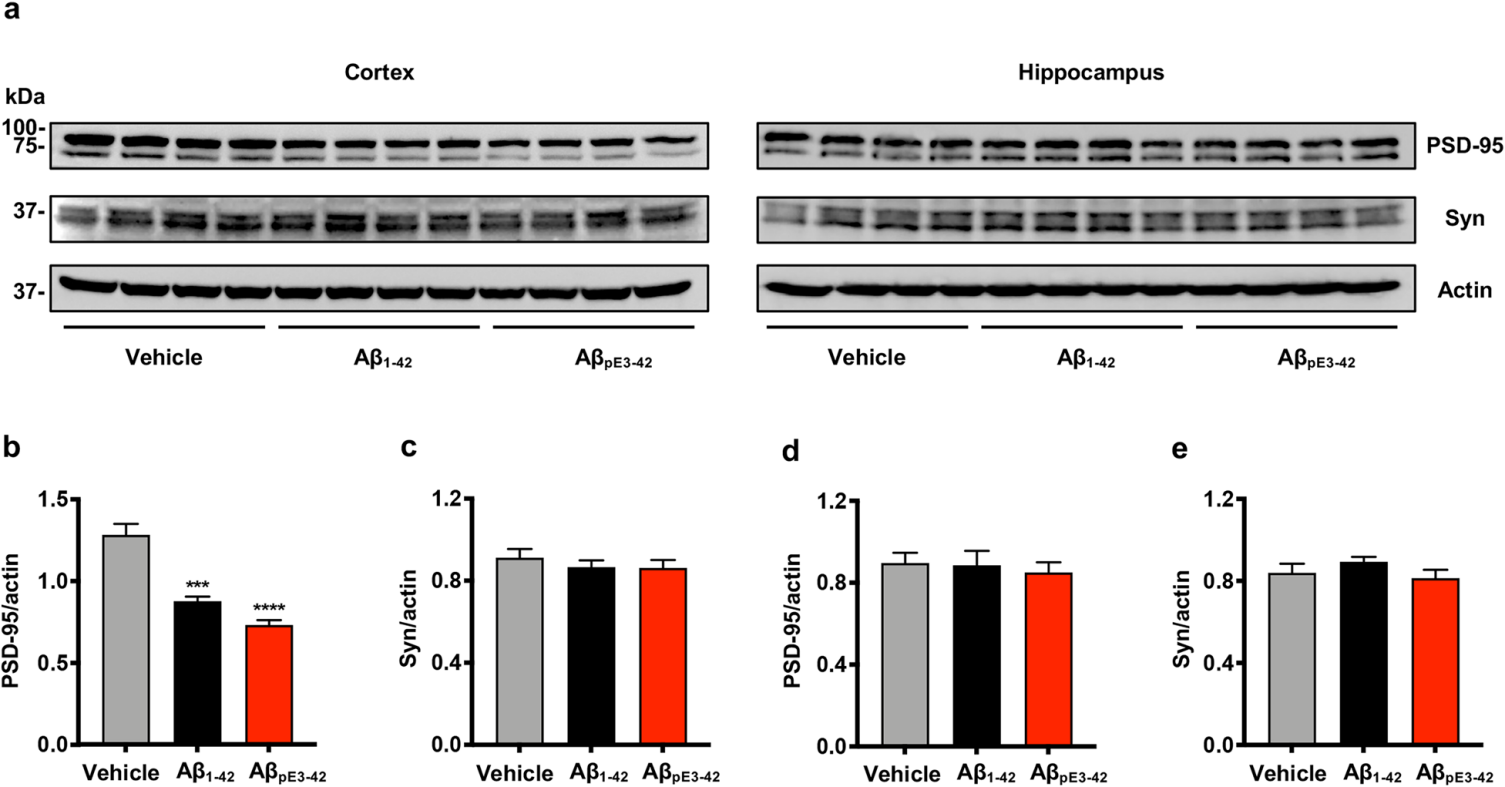
Aβ_pE3-42_-induced down-regulation of postsynaptic protein level in cortical lysate. (**a**) Western blot analysis of PSD-95 (85 kDa), synaptophysin (40 kDa), and β-actin (38 KDa) expression in hippocampal and cortical lysates of vehicles, Aβ_1-42_-infused mouse models, and Aβ_pE3-42_-infused mouse models. The original and uncropped full-membrane results with PSD-95, synaptophysin, and actin are presented in Supplementary Figure S5. Densitometry of PSD-95 and synaptophysin in (**b,c**) cortical and (**d,e**) hippocampal region. The intensities of blots were normalized to actin and statistically compared to vehicle (3 groups, n = 4 per group). One-way ANOVA analysis followed by Bonferroni’s *post-hoc* comparison tests were performed in all statistical analyses (***p < 0.001, ****p < 0.0001). The error bars represent S.E.M. PSD-95, Postsynaptic density protein 95; Syn, Synaptophysin; Actin, β-actin; Aβ_pE3-42_, Pyroglutamate amyloid-β_3-42_; Aβ_1-42_, Amyloid-β_1-42_, kDa, Kilodaltons.

As the behavior tests showed that synthesized Aβ_pE3-42_ disrupted cognitive function of ICR mouse models, we sacrificed the ICV-injected mice and prepared brain lysates to further observe the changes of synaptic protein levels by Aβ_pE3-42_ aggregates. Postsynaptic density protein 95 (PSD-95) regulates the synaptic strength of post-synaptic membrane, and synaptophysin supports synaptic vesicle endocytosis^29,30^. Both of proteins are highly related to the synaptic plasticity and widely used biomarkers for cognitive function. In order to examine such alterations in synaptic plasticity, we used a western blot method to analyze PSD-95 and synaptophysin expression levels in the brain lysates of Aβ_pE3-42_, Aβ_1-42_, and saline groups (Fig. 5a). All the quantification of blots were statistically compared to vehicle group (n = 4 per group). For the statistical analysis of the quantification, one-way ANOVA analysis followed by Bonferroni’s *post-hoc* comparison tests were performed. PSD-95 expression levels were decreased within the cortex region of Aβ_pE3-42_ (*p* < 0.0001) and Aβ_1-42_ groups (*p* < 0.001) compared to the saline groups (Fig. 5b). However, there was no change of synaptophysin levels among three groups (Fig. 5c). In the hippocampus region, both PSD-95 and synaptophysin expression levels were not significantly altered in Aβ_pE3-42_ and Aβ_1-42_ groups (Fig. 5d,e). All results from the western blot assay showed that the synthesized Aβ_1-42_ and Aβ_pE3-42_ oligomers suppress postsynaptic memory function in cortical region of the mice.

## Conclusion

In this study, we investigated a facile synthetic method of Aβ_pE3-42_ peptides and confirmed the amyloidogenic and pathologic properties of the synthesized peptides. In ThT assay and dot blotting analysis, we found that Aβ_pE3-42_ peptides rapidly accumulate into oligomers and fibrils. We further examined cytotoxicity of synthetic Aβ_pE3-42_ aggregates. Utilizing Aβ_pE3-42_-infused mouse models, we performed Y-maze and Morris water maze tests and observed synaptic dysfunction induced by synthetic Aβ_pE3-42_ oligomers. Furthermore, the expression level of PSD-95 was decreased in the cortical region of Aβ_pE3-42_-infusion mouse models compared to the vehicle groups. As Aβ_pE3-42_ aggregates are highly associated with AD, the presented synthetic method of Aβ_pE3-42_ will support pathological, therapeutic, and diagnostic investigation of the disorder.

## Methods

### Materials

Fmoc-Ala-OH, Fmoc-Arg(pbf)-OH, Fmoc-Asn(trt)-OH, Fmoc-Asp(OtBu)-OH, Fmoc-Cys(trt)-OH, Fmoc-Gln(trt)-OH, Fmoc-Glu(OtBu)-OH, Fmoc-Gly-OH, Fmoc-His(trt)-OH, Fmoc-Ile-OH, Fmoc-Leu-OH, Fmoc-Lys(Boc)-OH, Fmoc-Met-OH, Fmoc-Phe-OH, Fmoc-Ser(tBu)-OH, Fmoc-Tyr(tBu)-OH, and Fmoc-Val-OH were purchased from CEM (USA). Wang resin LS (0.22 mmol/g) was purchased from Advanced ChemTech (USA). Trifluoroacetic acid (TFA), triisopropylsilane, and 3,6-dioxa-1,8- octanedithiol (DODT), were purchased from TCI (Japan). Dimethylformamide (DMF), dichloromethane (DCM), and dimethyl sulfoxide (DMSO) were purchased from SAMCHUN chemical (Korea). Ether and acetonitrile were purchased from J.T Baker (USA). *N*,*N*′-diisopropylcarbodiimide (DIC), 4-dimethylaminopyridine (DMAP), ethyl cyanohydroxylminoacetate (Oxyma), piperidine, Boc-Pyr-OH, and ThT were purchased from sigma Sigma-Aldrich (USA). 96-well half area black plate was purchased from Corning (USA). 1X protease inhibitor cocktail was purchased from Roche Diagnostics (Switzerland). Pierce BCA protein assay kit was purchased from Thermo Fisher Scientific (USA).

### Animal preparation

Seven-week-old male ICR mice were purchased from Orient Bio Inc (Republic of Korea) and habituated for five days before the ICV injection. After the injection, behavior tests were performed.

### Ethical approval

All animal experiments were carried out in accordance with the National Institutes of Health (NIH) guide for the care, the use of laboratory animals (NIH Publications), and the ARRIVE guidelines. The research protocols were authorized by the Institutional Animal Care and Committee of Yonsei University (Seoul, Republic of Korea, IACUC-202103–1221-01).

### Safety statement

TFA is an acid, which severely irritates and burns the skin and eyes. Furthermore, breathing TFA will damage nose and throat. Thus, the protective clothes, googles, and gloves are required before treating TFA.

### Synthesis of Aβ_pE3-42_ peptides

Aβ_4-42_ peptide was synthesized through the previous Fmoc SPPS protocol^16^. For the first C-terminal alanine coupling, symmetric anhydride activation was utilized. We dissolved 1 mmol of DIC, 51 mg of 4-dimethylaminopydrine, and 2.2 mmol of Fmoc-Ala-OH dissolved in 2 mL of DMF/DCM (1:1 v/v) solution. In the solid-phase synthesis cartridge, DMF swelled Wang resin LS and the mixture were added and placed on a shaker for 1 h. After the complete symmetric anhydride activation, the rest of the amino acids were coupled by automated peptide synthesized (Liberty Blue, CEM, USA) with coupling reagents (1.0 M Oxyma pure and 1.0 M DIC solution in DMF) and deprotecting reagent (20% piperidine solution in DMF). After the synthesis of the truncated Aβ_4-42_ peptide, 1.1 mmol of Boc-Pyr-OH solution in DMF and coupling reagents (1.0 M Oxyma and 1.0 M DIC solution in DMF) were added to Aβ_4-42_ peptide on solid supports and reacted for 4 h. After the complete synthesis of Aβ_pE3-42_ peptide, 92.5% TFA cocktail (92.5:2.5:2.5:2.5 TFA/deionized water/triisopropylsilane/DODT, v/v/v/v) solution was added to the peptide bound resins and reacted for 4 h to cleave side chain protecting groups and Wang resin LS from the peptides. After cleavage of the peptides, we evaporated TFA solution by a rotary evaporator and added cold anhydrous ether (stored in −20 $^\circ$C) to yield the crude peptides. The peptides were isolated from the ether solution by centrifugation (3000 rpm, 15 min). The isolated mass and percent yield were 0.306 g and 71%, respectively.

### Purification of Aβ_pE3-42_ peptides

To purify and analyze the synthesized Aβ_pE3-42_ peptides, RP-HPLC was used with binary gradients of solvents A (0.1% TFA in deionized water) and B (0.09% TFA in acetonitrile). We utilized 5 μm biphenyl 250 × 21.2 mm column (Phenomenex, USA) with binary gradients from 20 to 55% of solvent B (6 mL/min, 38 min) and 5 μm biphenyl 250 × 4.6 mm column (Phenomenex, USA) with binary gradients from 10 to 90% of solvent B (0.8 mL/min, 38 min) for the purification and analysis of the peptides. The UV detection of all the peptides were conducted at 230 nm.

### Preparation of Aβ_1-42_ and Aβ_4-42_ peptides

To compare the amyloidogenic and neuropathological effects of Aβ_pE3-42_ peptide, Aβ_1-42_ and Aβ_4-42_ peptide were used as a control. Aβ_1-42_ and Aβ_4-42_ peptides were synthesized and purified utilizing the previously reported Aβ synthesis protocol^16^.

### ThT fluorescence assay

To monitor the aggregation propensity and quantify the β-sheet formation in Aβ_pE3-42_ aggregates, ThT fluorescence assay was performed. Aβ_pE3-42_ peptide was dissolved in DMSO (1 mM) and diluted with deionized water to make Aβ_pE3-42_ solution (25 μM). Each of sample was incubated for the various time range (1, 2, 4, 6, 8, 12, 24, 48, 72, 96 h) at 37 $^\circ$C. After the incubation, 25 μL of the incubated Aβ_pE3-42_ samples and 75 μL of ThT solution (5 μM ThT in 50 mM glycine buffer, pH 8.9) were added in a 96-well half area black plate. Fluorescence intensity of ThT ((λ_ex_ = 450 nm/λ_em_ = 485 nm) intercalated within Aβ_pE3-42_ aggregates was measured by using the microplate reader. To compare the aggregation propensities of Aβ_4-42_, Aβ_1-42_, and Aβ_pE3-42_, ThT assay was performed under the identical conditions as described above.

### Dot blotting analysis

To identify Aβ_pE3-42_ oligomers, each of Aβ_pE3-42_ samples (25 μM) was incubated for the time range (0, 2, 4, 6, 8, 12, 24, 48, 72, 96 h) at 37 $^\circ$C. 2 μL of each Aβ_pE3-42_ sample was loaded on a nitrocellulose membrane and blocked with a 5% skim milk solution for 1 h. After the blocking, we washed the membrane with TBST (tris-buffered saline with 0.1% Tween 20) for three times and incubated with primary antibody A11 (1:2000, Invitrogen, USA) for overnight at 4 $^\circ$C. After removing the primary antibody and washing the membrane with TBST for three times and added HRP-conjugated goat anti-rabbit secondary antibody (1:10,000, Bethyl Laboratories, USA) and incubated for 1 h at room temperature. Membranes were washed three times with TBST, and the proteins on the membranes were developed using ECL kit (Thermo Fisher Scientific, USA).

### Cell viability assays

HT 22 cell was purchased from the Korean Cell line Bank (Seoul National University, Republic of South Korea) and cultured in the Dulbecco’s Modified Eagle’s medium (DMEM) (Thermo Fisher Scientific, USA) with 10% foetal bovine serum and 1% penicillin. The cytotoxic effect of synthesized Aβ_1-42_, Aβ_4-42_, and Aβ_pE3-42_ was investigated using the previously reported MTT assay protocol^24^. Cultured HT22 cell (8 × 10^3^ cells/well) was seeded on a 96-well plate. Each Aβ variant (10 μM) was prepared in starvation medium (0.5% foetal bovine serum and 1% penicillin in DMEM) and treated on the plate for 24 h at 37 $^\circ$C. 15 μL of MTT reagent (Promega, USA) was added to each well and incubated for additional 4 h at 37 $^\circ$C. Finally, 100 μl of solubilization solution (Promega, USA) was added for 30 min at 37 $^\circ$C. The absorbance was measured at 570 nm.

### ICV injection

To examine cognitive deficits induced by Aβ_pE3-42_ oligomers, Aβ_pE3-42_ (10 μM in saline) was incubated for 6 h and 5 μL of Aβ_pE3-42_ oligomers were intracerebroventricular injected to ICR mouse using the previously reported ICV injection protocol^25^.

### Y-maze

For observing the short-term memory dysfunction initiated by Aβ_pE3-42_ oligomers, Y-maze test was performed. The maze was built with three arm entries (40 cm long, 10 cm wide, 12 cm high), which were symmetrically disposed at 120° angles. Each of mouse was placed at the edge of the arm entry and allowed to move around the maze for 8 min. After recording the experiment, the number of total arm entries and sequence of arm choices were analyzed. The percent alternation was calculated by the proportion of arm choices that differ from the previous two choices. After the trial, the maze was cleaned with 70% ethanol solution before the next trials.

### Morris water maze

Morris water maze tests was performed to monitor deficits of spatial learning function induced by Aβ_pE3-42_ oligomers. The maze contained a circular water tank (120 cm diameter, 25 ± 1 $^\circ$C) with the hidden quadrant, which was placed 1.5 cm below the water surface. The maze is equally divided into four quadrants (A,B,C,D) and three different shapes of cues are located on the top of quadrant B, C, and D to provide the direction for the mice. The hidden platform is placed on the middle of quadrant C and mice can survive if they reach the platform. The tank is filled with water with non-toxic white paint to prevent the chances that mice can see the hidden platform. We performed five days of training with four trials per day. In each trial, mouse was placed at the end of quadrant and allowed to swim and reach the platform for 60 s . After 60 s of trial, we took the mouse out of the maze and provided 10 min of resting time before the next trial. We measured escape latency, which is the average of the time to reach the platform during four trials. On the sixth day, we performed 60 s of probe test. Each mouse was placed at the edge of the opposite quadrant of the target zone. The hidden platform was removed and we analyzed crossing number, time in platform, time in the target quadrant, and swimming distance.

### Lysate preparation

After the behavior tests, we sacrificed all the mice to prepare brain lysates. We dissected the hippocampal and cortical regions of brains. All the brain lysates were homogenized in ice-cold RIPA buffer with 1X protease inhibitor cocktail. Homogenized brain lysates were incubated in ice for 20 min and collected the supernatants after the centrifugation at 14 000 rpm at$^\circ$C for 30 min. The protein concentrations in supernatants were quantified via Pierce BCA protein assay kit.

### Western blot

To analyze the alternation of synaptic protein marker levels, PSD-95 and synaptophysin levels, by Aβ_pE3-42_ aggregates, we conducted the western blot analysis. For the experiment, 15 μg of cortical and hippocampal brain lysates were loaded on the 12% gel and separated by sodium dodecyl sulfate polyacrylamide gel electrophoresis. We transferred the proteins from the gel to a nitrocellulose membrane and the membranes were blocked with a 5% skim milk solution for 1 h. After the blocking, we washed the membrane with TBST (tris-buffered saline with 0.1% Tween 20) for three times and incubated with primary antibody PSD-95 (1:2000, Invitrogen, USA) or synaptophysin (1:1000, MilliporeSigma, USA) for overnight at 4 $^\circ$C. After removing the primary antibody and washing the membrane with TBST for three times and added HRP-conjugated goat anti-mouse secondary antibody (1:10000, Bethyl Laboratories, USA) and incubated for 2 h at room temperature. Membranes were washed three times with TBST, and the proteins on the membranes were developed using ECL kit (Thermo Fisher Scientific, USA). For control of the protein loading levels, we used β-actin (1:2000, MilliporeSigma, USA).

### Statistical analysis

All graphical data were analyzed through GraphPad Prism 9.0 software, and statistical analyses were obtained from the one-way ANOVA followed by Bonferroni’s posthoc comparisons. The error bars represent the standard error of the mean (S.E.M).

### Equipment and settings

The scheme of Aβ_pE3-42_ synthesis was drawn by power point 16.56. Prism 9.0. was used to statistically analyze and visualize ThT fluorescence assay, cell viability assay, and behavior test results. FUSION Solo S and Image J Fiji 1.0. were used to visualize, quantify, and acquire dot blotting of Aβ_pE3-42_ aggregates with A11 and western blot of hippocampal and cortical lysates of Aβ_pE3-42_-infused mouse models, Aβ_1-42_-infused mouse models, and vehicles. All the quantification data were organized into graphical data with Prism 9.0.

## Supplementary Information

Supplementary Information.

## Data Availability

All data generated or analyzed during this study are included in this published article (and its Supplementary Information files).
