# Supplementary material for: Solid-phase synthesis and pathological evaluation of pyroglutamate amyloid-β3-42 peptide
Source: Sci Rep. 2023 Jan 10;13:505. doi: 10.1038/s41598-022-26616-x (PMC9831997; doi:10.1038/s41598-022-26616-x)
Supplement: Supplementary file 1 — Supplementary Information. [file 41598_2022_26616_MOESM1_ESM.pdf]

Supplementary Information

Solid-phase synthesis and pathological evaluation of pyroglutamate amyloid-β_{3-42} peptide

Authors

Illhwan Cho[1,2], HeeYang Lee[1,2], Donghee Lee[1,2], In Wook Park[1,2], Soljee Yoon[1,2,3], Hye Yun Kim[1,2,*], and YoungSoo Kim[1,2,3,4,*]

Affiliations

[1]Department of Pharmacy, College of Pharmacy, Yonsei University, Incheon 21983, Republic of Korea

[2]Yonsei Institute of Pharmaceutical Sciences, College of Pharmacy, Yonsei University, Incheon 21983, Republic of Korea

[3]Department of Integrative Biotechnology and Translational Medicine, Yonsei University, Incheon 21983, Republic of Korea

[4]Yonsei-POSTECH Campus, Pohang University of Science and Technology (POSTECH), Pohang, Gyeongbuk 37673, Republic of Korea

*Corresponding Authors

Hye Yun Kim; hyeyunkim@yonsei.ac.kr; Tel.: +82-32-749-4526

YoungSoo Kim; y.kim@yonsei.ac.kr; Tel.: +82-32-749-4523

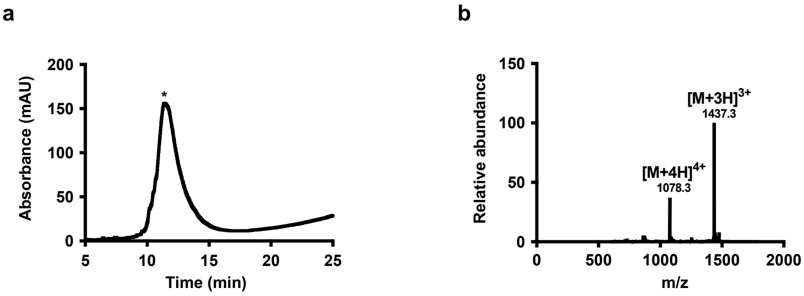

Supplementary Figure S1. Reverse phase-high performance liquid chromatography and electrospray ionization-mass spectrometry analysis of synthesized A$\beta_{pE3\text{-}42}$ peptide. (**a**) UV peak of purified A$\beta_{pE3\text{-}42}$ peptide. UV detection was conducted at 230 nm. (**b**) Asterisk (*) represents A$\beta_{pE3\text{-}42}$ at 12.4 min with exact mass spectrum. mAU, Milli-absorbance units; min, Minutes; m/z, Mass to charge ratio.

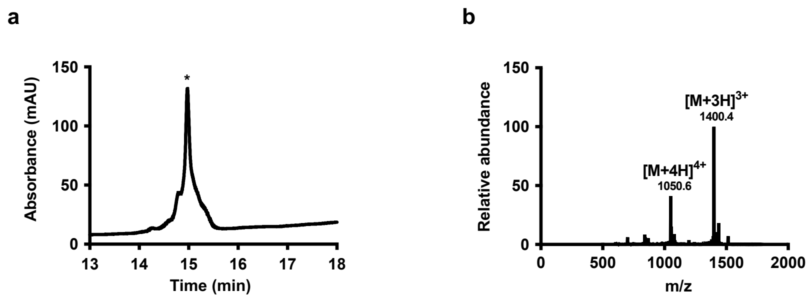

Supplementary Figure S2. Reverse phase-high performance liquid chromatography and electrospray ionization-mass spectrometry analysis of synthesized Aβ$_{4-42}$ peptide. (**a**) The UV peak of purified Aβ$_{4-42}$ peptide. UV detection was conducted at 230 nm. (**b**) Asterisk (*) represents Aβ$_{4-42}$ at 15.0 min with exact mass spectrum. mAU, Milli-absorbance units; min, Minutes; m/z, Mass to charge ratio.

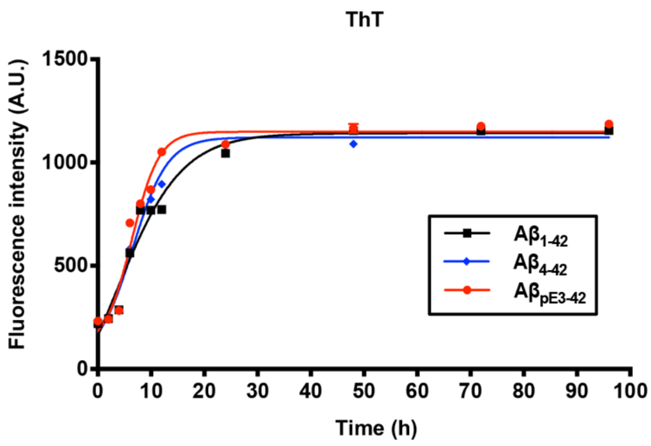

Supplementary Figure S3. ThT fluorescence assay for monitoring aggregation of Aβ variants. 25 µM of Aβ variants (Aβ$_{1\text{-}42}$, Aβ$_{4\text{-}42}$, and Aβ$_{pE3\text{-}42}$) were incubated for the time points (0, 2, 4, 6, 8, 10, 12, 24, 48, 72, 96 h) at 37˚C. Each of incubated Aβ variant was interacted with ThT (5 µM) and the fluorescence intensity was measured by the fluorescence reader. The error bars represent S.E.M. Aβ$_{1\text{-}42}$, Amyloid-β$_{1\text{-}42}$; Aβ$_{4\text{-}42}$, Amyloid-β$_{4\text{-}42}$; Aβ$_{pE3\text{-}42}$, Pyroglutamate amyloid-β$_{3\text{-}42}$; h, Hours; ThT, Thioflavin T.

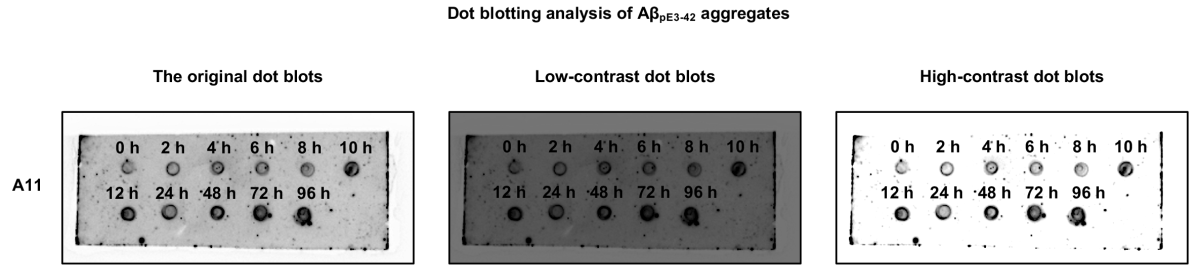

Supplementary Figure S4. The original and uncropped full-membrane images of dot blotting assay with A11. Utilizing anti-oligomer polyclonal A11, dot blot analysis was performed to detect oligomeric forms of incubated $A\beta_{pE3-42}$ samples. h, Hours; $A\beta_{pE3-42}$, Pyroglutamate amyloid-β_{3-42}

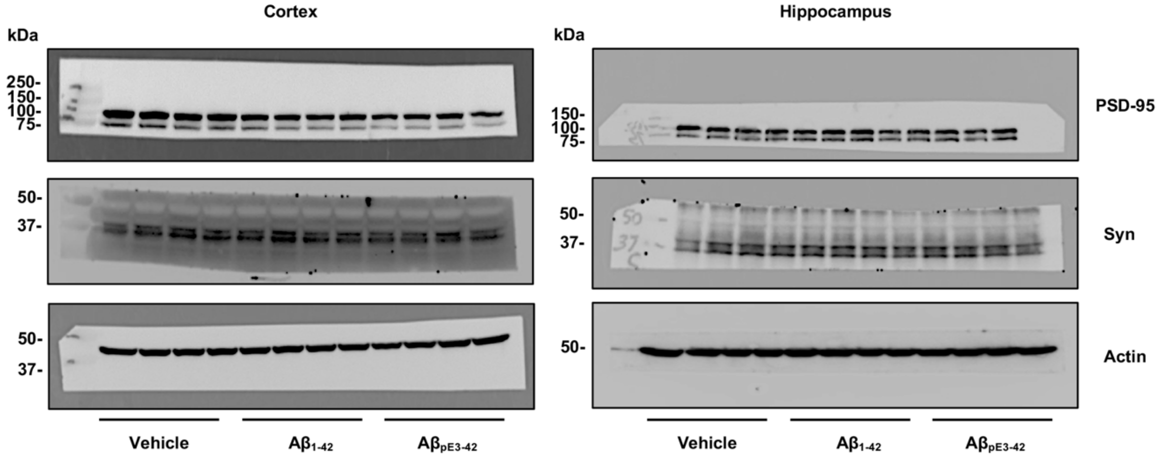

Supplementary Figure S5. The original and uncropped full-membrane images of western blot analysis. Utilizing anti-PSD-95 polyclonal antibody, anti-synaptophysin monoclonal antibody, and anti-β-actin monoclonal antibody, western blotting assays were performed. The uncropped and full-membrane images including postsynaptic density protein 95, synaptophysin, and β-actin in cortical and hippocampal regions of vehicles, $A\beta_{1-42}$-infused mouse models, and $A\beta_{pE3-42}$-infused mouse models. Left lane of each membrane represents the molecular size marker. PSD-95, Postsynaptic density protein 95; Syn, Synaptophysin; Actin, β-actin; $A\beta_{pE3-42}$, Pyroglutamate amyloid-β_{3-42}; $A\beta_{1-42}$, Amyloid-β_{1-42}; kDa, Kilodaltons.
